# Texture of Hot-Air-Dried Persimmon (*Diospyros kaki*) Chips: Instrumental, Sensory, and Consumer Input for Product Development

**DOI:** 10.3390/foods9101434

**Published:** 2020-10-10

**Authors:** Rebecca R. Milczarek, Rachelle D. Woods, Sean I. LaFond, Jenny L. Smith, Ivana Sedej, Carl W. Olsen, Ana M. Vilches, Andrew P. Breksa, John E. Preece

**Affiliations:** 1 United States Department of Agriculture—Agricultural Research Service, Western Regional Research Center, Healthy Processed Foods Research Unit, 800 Buchanan Street, Albany, CA 94710, USA; rachelle.woods@usda.gov (R.D.W.); ivana.sedej@usda.gov (I.S.); carl.olsen@usda.gov (C.W.O.); ana.vilches@usda.gov (A.M.V.); andrew.breksa@usda.gov (A.P.B.); 2 Department of Food Science & Technology, University of California, One Shields Avenue, Davis, CA 95616, USA; silafond@ucdavis.edu; 3 United States Department of Agriculture—Agricultural Research Service, National Clonal Germplasm Repository, One Shields Avenue, Davis, CA 95616, USA; jenny.smith@usda.gov (J.L.S.); john.preece@usda.gov (J.E.P.)

**Keywords:** persimmon (*Diospyros kaki*), Texture Profile Analysis (TPA), shear test, sensory evaluation, consumer, hot-air-drying, value-added product

## Abstract

Persimmon (*Diospyros kaki*) is an underutilized tree fruit. Previous studies have shown the feasibility of making a hot-air-dried, chip-style product from persimmon. However, the texture of this type of product has not been explored or connected to consumer preference. Thus, for dried samples representing 37 cultivars, this study aimed to (1) predict trained sensory panel texture attributes from instrumental measurements, (2) predict consumer liking from instrumental measurements and sensory texture attributes, and (3) elucidate whether astringency type affects dried product texture. Partial least-squares regression models of fair-to-good quality predicted all measured sensory texture attributes (except Tooth Packing) from instrumental measurements. Modeling also identified that consumer preference is for a moist, smooth texture. Lastly, while astringency type has significant (*p* < 0.05) effects on several individual texture attributes, astringency type should not be used a priori to screen-in or -out persimmon cultivars for processing into a hot-air-dried product.

## 1. Introduction

Persimmon (*Diospyros kaki*) is an orange-skinned, subtropical tree fruit grown in several regions worldwide but largely unknown in the United States outside of farmers’ markets and home growers in the State of California. To encourage more widespread consumption of persimmons, researchers have endeavored to develop a value-added product (a hot-air-dried persimmon “chip”) that is shelf-stable and easily produced. Several groups [1,2,3,4,5,6] have previously conducted exploratory studies of sliced, hot-air-dried persimmon chips. However, in-depth information on the texture of these products is lacking. Gathering texture information to inform product development can be very time- and resource-intensive, so one aim of the present study was to determine how well instrumental texture measurements (relatively low-time and low-resource) would correlate with sensory texture attributes (moderate-time, moderate-resource). In turn, another aim was to determine which texture measurements (both instrumental and sensory) could best predict consumer liking of the products, given that consumer studies typically have the highest time and resource requirements. This information would be of use to other teams developing dried fruit products.

The Texture Profile Analysis (TPA) double compression test, originally developed by Szczesniak et al. [7,8], is often the instrumental method of choice for studies such as this. The TPA method yields actionable information when the product under testing is spatially uniform in composition. Persimmon chips, however, have two distinct physical features: the flesh (interior) and the skin (exterior). When one bites through the dried cross-section of a persimmon, the fruit’s skin can be a prominent feature, and the properties of the skin would not be captured in the results of TPA performed on a disk excised from the flesh portion of the sample. Thus, an additional instrumental texture measurement is needed to fully characterize this product. Greve et al. [9] used multiple instrumental variables derived from a variety of five test probes (three-point bending, needle, 2 mm/6 mm cylinder compression, and small blade shear) to predict the sensory texture attributes for commercial snack bars. Of the tested probes, the blade exhibited the highest overall predictive ability, showing a validation coefficient determination greater than 0.8 for all the sensory attributes evaluated (Hardness, Crispness, Brittleness, Hand-Evaluated Hardness). Thus, both TPA and a shear cutting method were employed in the present study.

Instrumental and sensory measurements of texture performed on a macroscopic level reflect the status of the microstructure of the fruit or vegetable; this has been shown for crops such as apples [10], olives [11], onions [12], and potatoes [13]. For crops destined for drying, both the composition [14,15] and morphology [15,16,17,18,19,20] of the plant cell wall play important roles in the final product’s microstructure (and thus the overall texture). Certainly, the cell water status/turgor pressure [13], the maturity level of the raw fruit [21,22], and the drying process conditions [23,24,25,26] also influence the dried product microstructure and macro-texture, but these factors were held constant in the present study, in that all persimmon samples were harvested at the same maturity level and hot-air-dried under identical conditions, with the final products having nearly identical (and very low) moisture contents. 

Even with all these variables held constant, the raw fruit microstructure can be expected to vary among cultivars [16,21,27,28]; hence, it is important to consider the effect of cultivar on the dried product texture. Persimmon cultivars can be classified into multiple types based on their astringency at harvest. Nonastringent cultivars are ready to eat immediately upon harvest, i.e., can be eaten like an apple. If the fruit of pollination variant (referred to as simply “variant” hereafter) cultivars are pollinated in the spring (and thus have seeds upon maturation), the flesh color will change from orange to brown and the fruit will be palatable immediately upon harvest. In contrast, a deastringency process must be applied to astringent and unpollinated (orange-flesh) variant cultivars in order for these persimmon types to become palatable. While there is one study in the literature that addresses whether/how the astringency type affects the texture of fresh persimmons [29], there is no published information addressing how astringency type (and variant-brown vs. variant-orange phenotype) might affect the texture of hot-air-dried persimmon products. Thus, the final goal of this work was to address this knowledge gap and give growers/processors some guidance for cultivar selection for hot-air drying. This goal is in support of a larger project on enhancing the marketability of persimmons grown in the U.S. State of California; other aspects of the larger project have been reported in our previous works [1,2,30]. 

## 2. Materials and Methods 

### 2.1. Persimmon Samples

Forty-eight persimmon samples, each consisting of ~200 fruit, were harvested in fall 2016 (Table 1). The samples included 37 cultivars: 12 astringent, 9 nonastringent, 15 variant, and 1 unknown (commercial retail sample). Within the variant astringency type, samples were further designated as either “variant-orange” or “variant-brown” phenotype based on a visual inspection of photographs of the dried material. If more than 50% of the surface area of the dried slices of a given sample had predominantly orange flesh color, that sample was designated “variant-orange”, while if more than 50% was predominantly brown, that sample was designated “variant-brown”. Due to uneven pollination of the persimmon trees, it was possible for a single cultivar to be variant-orange for one sample and variant-brown for another sample. (Indeed, this occurred for cultivar ‘Giombo’, whose first harvest was variant-orange and whose second harvest was variant-brown; this is addressed further in Section 3, Results and Discussion.) The implications of uneven and/or unpredictable pollination for the texture of hot-air-dried variant persimmon cultivars are quite profound. Persimmon growers strongly desire the flowers on their trees to either be completely pollinated or completely unpollinated and can face substantial economic losses if the desired level of pollination is not achieved. Thus, one strength of the sample set used in this work was the inclusion of variant cultivars exhibiting both the orange/unpollinated and brown/pollinated phenotypes. 

The persimmon samples were acquired from 3 sources: the United States Department of Agriculture—Agricultural Research Service National Clonal Germplasm Repository for Fruit & Nut Crops (USDA-ARS NCGR, Davis, CA, USA) (denoted as “R” in Table 1 and throughout this work), a commercial nursery (L.E. Cooke, Co., Visalia, CA, USA) (denoted as “C-N”), and a commercial retail store (denoted as “C-S”). For some of the R cultivars, there was enough fruit available to collect multiple samples throughout the season; in these cases, the sample harvests were spaced apart by a minimum of 12 days. Persimmons were hand-harvested when commercially ripe, i.e., having at least some exterior orange skin color. The R and C-N persimmon samples were packed directly into boxes lined with molded plastic cushioning trays. The boxes of fruit were transported to the USDA-ARS laboratory in Albany, Calif. within 24 h of harvest. The C-S sample (already in dried form) was purchased from a grocery store in El Cerrito, Calif. in February 2017. 

### 2.2. Drying Method

Full details of the hot-air drying method are given in our previous work [1]. Briefly, prior to drying, freshly-harvested fruit were stored for an average of 7 days (range of 3 to 15 days). Following current best practices [31], storage temperature was dependent on astringency type: 18 °C (64 °F) for nonastringent cultivars and 2 °C (36 °F) for variant and astringent cultivars. Persimmons from the R and C-N samples were sliced into circular sections of 5 mm in thickness, and the slices were dried at 52 °C (125 °F) for 18 h in a commercial dehydrator (Model 2924T, Excalibur Dehydrator, Sacramento, CA, USA). All slices had a “wagon wheel” shape, with skin only present around the circumference of each slice; slices from the blossom- and stem-ends of the fruit were not used in this study. The dried slices (chips) were stored at ambient temperature in sealed metallized polyester film pouches until they were used for the instrumental, trained sensory panel, and consumer panel analyses in Spring 2017. The C-S sample was already in circular form; it was stored in its original packaging until needed for analysis. 

### 2.3. Instrumental Measurements

Measurements were performed on the 48 samples of dried persimmons in February through April 2017. To ensure that all samples had remained adequately dry after several months of storage, 3 dried persimmon slices were randomly selected from each sample bag for a check of moisture content and water activity. Moisture content was determined gravimetrically, by drying the ground samples in a vacuum oven at 70 °C for 24 h. Water activity was measured via the dew point method at 25 °C in an AquaLab CX-2 water activity meter (Decagon Devices, Pullman, WA, USA).

For the instrumental texture measurements, 6 dried persimmon slices were randomly selected from each sample; 3 slices were subjected to TPA and 3 to the shear test. The mean values across the triplicate measurements were reported. Both tests were conducted on a TA-XTPlus100 Texture Analyzer (Stable Micro Systems Ltd., Godalming, UK) equipped with a 100 kg load cell.

#### 2.3.1. Texture Profile Analysis (TPA)

Using a cork borer, a 7 mm disk was excised from the persimmon slice from tissue midway between the skin and center axis (and away from any seed compartments, if present). Using a TA-11ss probe (stainless steel cylindrical probe 25.4 mm in diameter), the disk was subjected to a standard TPA 2-cycle compression protocol, which has been well-described in the literature for similar products [32,33,34]. The probe speed was 1.67 mm/s, and the disks were compressed to 50% strain. The derived measurements from TPA are summarized in Table 2. 

#### 2.3.2. Shear Test

A custom-made stainless steel blunt shear blade of 1 mm thickness was used for the shear test. The blade cut through the sample along a 15 mm line, with the persimmon skin at either end of the line and flesh in between. The 15 mm distance was chosen for 2 reasons: (1) it is a reasonable distance to approximate human incisors biting through a circular chip-style object and (2) the smallest persimmon chip diameter observed in this study was ~20 mm, and an achievable shear test distance needed to be guaranteed for all the samples. The blade cut through the chip at the designated line at 1.67 mm/s, and a stopping distance of 150% of the chip height was used to ensure that the blade had gone completely through the chip. The resulting force versus distance trace had one major peak, with a varying number of minor peaks on the downstroke. The 3 measurements derived from this trace are summarized in Table 2.

### 2.4. Trained Panel Sensory Evaluation

Descriptive sensory analysis took place in parallel with the instrumental texture measurements described in the previous section. The organization, training, and conduction of the sensory panel are described in detail in our previous work [1]. Here, we will focus on the texture component of the sensory analysis. 

Nine adult panelists (seven female, two male, aged 35–65) were recruited from the USDA-ARS laboratory (Albany, CA, USA). Four panelists had experience evaluating dried persimmons during the previous year; the remainder were included in the panel based on their availability for the full 7 week course of the study and their demonstrated basic sensory acuity. All panelists participated in at least 4 1-h training sessions. The panelists were trained on 8 texture attributes, in addition to the taste, aftertaste, and flavor attributes, which were used for other portions of the larger study described in Milczarek et al. [1]. The texture attributes were initially gleaned from a reference work [35] and had been refined with panelist input the previous year. The panelists rated the texture attributes on a 15 cm unstructured line scale with anchors. Definitions and standards for the sensory texture attributes are listed in Table 3. 

The trained panelists evaluated the dried persimmon samples in isolated booths under ambient fluorescent lighting. The panelists were given one whole chip to assess Roughness, Moistness, Hardness, Crispness, Skin Toughness, and Fibrousness and one wedge (1/4 to 1/3 of a chip; size adjusted to ensure equal mass among the samples) to assess Chewiness and Tooth Packing. Panelists received 4 samples evaluated in triplicate (i.e., 12 products per session. Product presentation order was randomized, and the persimmon chips were presented monadically in black soufflé cups labeled with 3 digit random codes. The panelists were instructed to cleanse their palates between samples with filtered water and unsalted water crackers, which is a palate-cleansing approach recommended for high-astringency foods [36]. The panelists evaluated samples 2 days a week for 7 weeks. 

### 2.5. Consumer Test

The basic structure of the consumer test was a balanced incomplete block design (BIBD), identical to that described in our previous work [1]. The BIBD approach has been used in the past for gathering consumer data when the number of study samples is large but the number of samples each panelist can be reasonably expected to evaluate is quite small [37,38]. In brief, a subset of 25 of the dried persimmon samples were evaluated by 150 consumers for overall liking. Each consumer evaluated 5 of the 25 samples in a rank-rating task; only the rating (hedonic score of 1 (dislike) to 15 (like)) was considered in the present study. A similar rank-rating approach was used by Cordonnier and Delwiche [39] for consumer evaluation of lemonades; in that case, the researchers found that rank-rating and a 9-point hedonic scale seemed to be equivalent in terms of differentiation of samples and statistical power. In the present study, after providing their overall liking score, each consumer was asked to describe the color, texture, and flavor of their most-liked sample in free-response format. Lastly, each consumer provided basic demographic information and data about consumption frequency of fresh persimmons and dried fruit. The exact wording of the questions can be found in the consumer response sheet, available as Supplementary File S1.

The consumer test was conducted at the USDA-ARS Western Regional Research Center laboratory (Albany, CA, USA) on a single day in March 2017. The venue was a quiet section of an employee break room/lunchroom. The participants included laboratory employees (81%) and a group of visiting school students (19%). This type of convenience sample of subjects is generally not ideal for a consumer test [40,41]. However, resource limitations in the project precluded an off-site test, and the panel composition nonetheless had several favorable aspects. Cardinal et al. [41] have pointed out that convenience sampling of so-called consumers is particularly problematic if the panelists have formal food science knowledge and/or deep technical knowledge of the product category. In this study, all of the consumers were unware of the purpose of the study and the nature of the samples. There was no overlap between the membership of the consumer group and that of the trained panel. The laboratory employees in the consumer panel worked in a variety of roles (including clerical and non-food-related technical), and the students had no training in food science. The consumer group was diverse in terms of both gender and age: 61% female/39% male and age range from “10–18 years old” to “70 years and over”. In terms of fresh persimmon consumption during the usual harvest season, the group was skewed toward low-or-no consumption of fresh persimmons, with 55% selecting “never” or “less than once a month” for this item. However, for dried fruit in general, a strong majority (77%) of the group were moderate consumers of dried fruit (on its own, i.e., not in a trail mix, cereal, etc.), reporting consumption rates between “less than once a month” and “1–2 times a week”. Hence, the consumers represented a reasonable target audience for a dried persimmon chip product, and assembling a representative consumer panel is important for consumer study design [40]. Nonetheless, the authors acknowledge the limitations of the consumer portion of this study and do not recommend that the consumer results be extrapolated to the population at large. 

Due to the restrictions on both the panel size and block size in the experiment design, only 25 of the 48 samples could be included in the consumer study. The researchers chose this subset of 25 samples based on the following criteria:

- first harvests of R cultivars that had not been evaluated by consumers in our [1] previous consumer tests (14 samples);
- cultivars from source C-N that had not been evaluated by consumers in our previous consumer tests (3 samples);
- cultivars known to have high consumer preference based on the results of our previous tests (7 samples);
- C-S retail sample (1 sample).

The samples used in the consumer test are marked in Table 1 with a ‘+’ symbol. 

### 2.6. Statistical Analyses

All statistical analyses were conducted using JMP (Version 9.0.1, SAS Institute, Inc., Cary, NC, USA). Partial least-squares regression (PLS) was used to construct models predicting the sensory attributes from instrumental measurements and models predicting the consumer liking score from both instrumental measurements and sensory attributes. To validate the models, leave-one-out cross-validation was performed and the root-mean-square error (RMSE) of cross-validation reported. (Well-performing models will have a low RMSE.) In addition, quality of the models was assessed based on the adjusted R^2^ value for the linear fit of predicted versus measured values. The assessments were as follows: “Poor”—adjusted R^2^ less than 0.3, “Fair”—0.3 to 0.6, “Good”—0.6 to 0.9, “Excellent”—above 0.9. 

As part of the method of PLS, the relative importance of each variable in a model is given by its variable importance in projection (VIP) score. Variables with VIP scores greater than a given threshold contribute significantly to the model, while variables with VIP scores less than the threshold do not, that is, they could be left out without reducing the performance of the model. Since the average of the squared VIP scores equals 1, the threshold is often set at 1.0. However, when the proportion of relevant predictors is low and the magnitude of correlation among predictors is high, the appropriate cutoff value is required to be greater than 1.0 and can be as high as 1.21 [42]. Since our preliminary work [43] revealed that several of the instrumental measurements and sensory attributes were highly correlated with each other, a VIP threshold of 1.2 was chosen for the present study.

For the models predicting consumer liking from the texture measurements, PLS VIP score was used as a variable selection method. Ordinary least-squares (OLS) models—termed “sparse” models—were constructed from only those variables whose PLS VIP scores were greater than 1.2. The RMSE of cross-validation and adjusted R^2^ of predicted versus actual liking were again calculated and used to assess the quality of the OLS models. The coefficients of the OLS models were also reported, primarily to clarify the direction of a variable’s influence (positive coefficient indicates increasing the value of the variable would increase consumer liking, negative coefficient indicates decreasing the value of the variable would increase consumer liking). 

An analysis of variance (ANOVA) was performed to determine the effect of the 4 persimmon astringency types/subtypes (astringent, nonastringent, variant-brown, variant-orange) on the mean values of the instrumental measurements and sensory attributes. An ANOVA probability value of *p* < 0.05 was chosen to indicate a significant difference among 2 or more astringency types/subtypes. The post hoc analysis of Tukey’s Least Significant Difference (Tukey’s LSD) was applied to elucidate which astringency types/subtypes differed from each other; a difference of *p* < 0.05 between 2 levels was considered significant. It is noted that since the provenance (cultivar, harvest date, astringency type, etc.) of the commercial retail sample was unknown, this sample could not be included in the ANOVA, but it was included in all the other analyses.

## 3. Results and Discussion

### 3.1. Moisture Content and Water Activity of Persimmon Chips

The thorough drying approach (52 °C for 18 h) used in this study was selected to ensure that all dried persimmon samples would be well below a water activity (a_W_) of 0.65. Thus, it is not surprising that the majority of the samples (45 of the 47 samples dried by the researchers) had very low mean moisture content and a_W_ values and that these values were in a narrow range: 6.79% (standard deviation (s.d.) = 0.93%) for the wet basis moisture content and 0.3691 (s.d. = 0.0394) for a_W_. These values are reasonable when comparing the persimmon chips with a similar product: apple chips. While the moisture content of the persimmon chips in this study was below that reported for apple chips (16.7%), a_W_ was slightly higher for the persimmon chips than it was for the apple chips (approximately 0.3) [44].

With respect to moisture content and a_W_, there were three outliers that could not be discarded. The chips from the second harvest of cultivar ‘Nui Nai’ had a moisture content of 11.14% (s.d. = 1.76%) and a a_W_ of 0.6083 (s.d. = 0.0752); the chips from the third harvest of cultivar ‘Mikatani Gosho’ had a moisture content of 12.70% (s.d. = 3.41%) and a a_W_ of 0.6253 (s.d.= 0.1044). The store-bought dried persimmon sample C-S (drying method, cultivar, and provenance otherwise unknown) had a moisture content of 10.89% (s.d. = 2.91%) and a a_W_ of 0.4880 (s.d. = 0.0071). For ‘Nui Nai’ and ‘Mikatani Gosho’, it should be noted that the unusually high moisture content/a_W_ occurred only for the second and third harvests, respectively, of these cultivars; the values for the same cultivars at earlier harvest dates were within the range for the rest of the set of samples. At the late-season harvests, the fruit were over-ripe—likely beyond the ripeness level that would be used for dried chips (since fruit that are soft are difficult to slice). These samples were nevertheless included in the analyses for this work, since they were dried, packaged, stored, and analyzed in the same way as the rest of the samples. Sample C-S, too, was included in all analyses except the ANOVA for astringency type (described later, in Section 3.4). 

### 3.2. Predicting Sensory Attributes from Instrumental Measurements

For the hot-air-dried persimmon chips, instrumental measurements can be used to predict some sensory attributes. Table 4 summarizes the model diagnostics and VIP scores for prediction of the 8 sensory attributes from the 8 TPA measurements and 3 shear test measurements. 

While none of the models could be considered “Excellent”, “Good” predictive models (low RMSE and high R^2^) can be constructed for the sensory attributes Chewiness, Crispness, Hardness, and Skin Toughness. “Fair” models can be constructed for Fibrousness, Moistness, and Roughness, and a “Poor” model could be constructed for Tooth Packing. The latter result is in line with our findings from dried persimmons from the previous year [43] and is an expected result, since Tooth Packing is evaluated after expectoration, which is not a physical situation that was simulated with either of the two instrumental methods used in this study.

In the models predicting sensory attributes from the two instrumental methods, the shear test generally yielded more predictive information than did TPA. This is evidenced by the fact that the VIP scores for the shear test were generally higher than those for TPA. In fact, none of the TPA measurements of Hardness 1, Cohesiveness, Springiness, or Chewiness had VIP scores greater than the threshold of 1.2.

There are some specific cases of note from this analysis:

1. The sensory attribute Hardness was better predicted by the two TPA Compressive Energy measurements than it was from either TPA Hardness measurement. This suggests that sensory evaluators are considering more than just the single highest peak force when making their assessment of Hardness and is in line with the equipment manufacturer’s caution that “(r)esearchers should understand that consumers’ judgments of Hardness can be more nuanced than a simple peak force metric and in some instances might be able to attain better correlations with the downstroke area of work.” [45].
2. The single highest VIP score was for the shear method measurement Peak Count predicting the sensory attribute Crispness; this result is in agreement with the psychophysical model of how crispness is perceived by humans [46].
3. The TPA measurement Chewiness did not, in fact, predict sensory Chewiness very well. The TPA measurements Compressive Energy 1, Hardness 2, Compressive Energy 2, and Resilience, and the shear method measurement Cutting Force all had VIP scores greater than 1.2, while TPA Chewiness had a VIP score of 0.972. The non-utility of TPA Chewiness in predicting sensory Chewiness is surprising, since there is a strong correlation between the two for snack bars [32].

Readers who are interested in additional insights into the relationships among the instrumental and sensory texture variables may refer to the Principal Components Analysis (PCA) described in Appendix A. 

### 3.3. Predicting Consumer Liking from Instrumental Measurements and Sensory Attributes

While it is instructive and time-saving to be able to predict sensory texture attributes from instrumental measurements, ultimately, consumer preference is the most desired piece of information about a new product. Table 5 shows the results of the PLS modeling of consumer liking based on both instrumental measurements and sensory texture attributes as well as each category individually. 

Since texture is certainly not the only aspect of a product that drives consumer liking, it is not surprising that the PLS models predicting consumer liking solely from texture measurements are “Fair”, at best. Adding measurements of taste and flavor to the models would improve their predictive ability, but this is beyond the scope of this study and is discussed in our work on persimmon chips from the previous harvest season [1]. Nonetheless, the exercise of modeling consumer liking solely from texture attributes helps to narrow down which of these attributes are most important to consumers. Table 6 lists the coefficients of the OLS models constructed using measurements/attributes that had VIP scores greater than 1.2 in the PLS models. 

In the highest-resource testing case (in which both instrumental and trained sensory panel measurements are available), 6 of the 8 sensory attributes—all except Roughness and Moistness—were predictive of overall consumer preference, and one instrumental measurement (shear Peak Count) contributed as well. So, a shear test coupled with and abbreviated-attribute-list sensory evaluation would be sufficient to obtain “Fair” predictive ability for consumer liking. In the medium-resource case (only an instrumental test or a trained sensory panel test can be performed), a comparison of the RMSE and adjusted R^2^ values in Table 6 indicates that texture evaluation by a trained sensory panel yields a more accurate prediction of consumer preference than do instrumental measurements alone, so the sensory panel would be preferred. In fact, in this theoretical sensory panel, only 2 attributes—Fibrousness and Chewiness—need to be evaluated. Lower values of these attributes are preferred by consumers; see the negative coefficients for these attributes in the “Sensory Only—Sparse” row of Table 6. Even so, instrumental measurements, especially those from the shear test, can provide some predictive ability with even lower time and resource requirements than organizing, training, and carrying out a sensory panel. In fact, the diagnostics for the “Instrumental Only—Sparse” and “Shear Peak Count Only” OLS models (Table 6) are very similar, indicating that the Peak Count measurement from the shear test would be the one measurement of choice in a very resource-limited texture testing situation. Lower values of this metric would be preferred by consumers. 

The importance of the texture of the skin of the fruit was revealed in this portion of the study, in that the shear test (measures both skin and flesh) generally gave more predictive results of consumer liking than did TPA (measures flesh only). Several consumer panelists provided free-response comments about the skin of the samples; testing acceptance of peeled and unpeeled chips would be a logical next step in the product development process. In addition, the synthesis of the instrumental, sensory, and consumer data indicates that targeting a softer, moister, dried-fruit product derived from persimmons will be a more desirable route than the crispy, chip-like product the development team originally envisioned.

### 3.4. Effect of Astringency Types

#### 3.4.1. Comparison on As-Dried Basis

**Figure 1:**
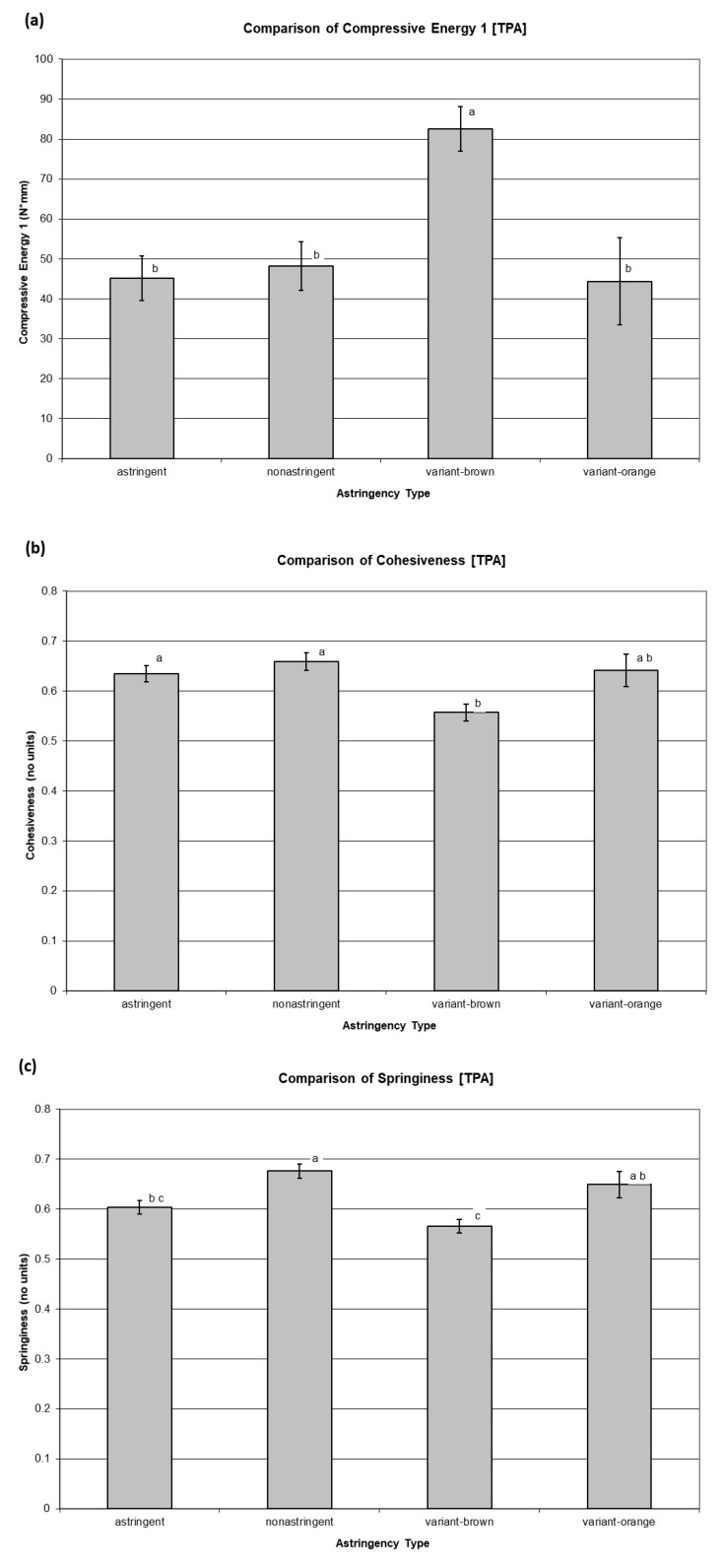

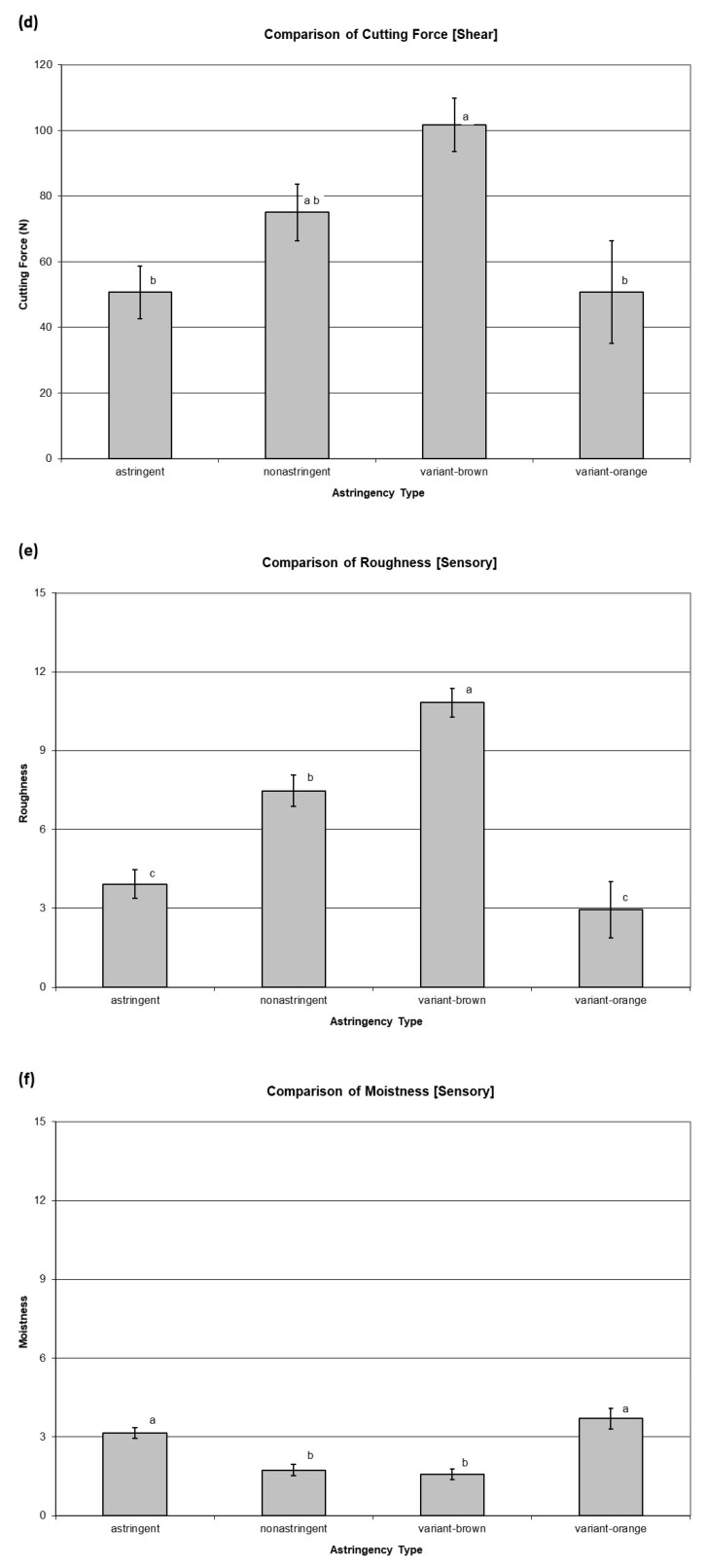

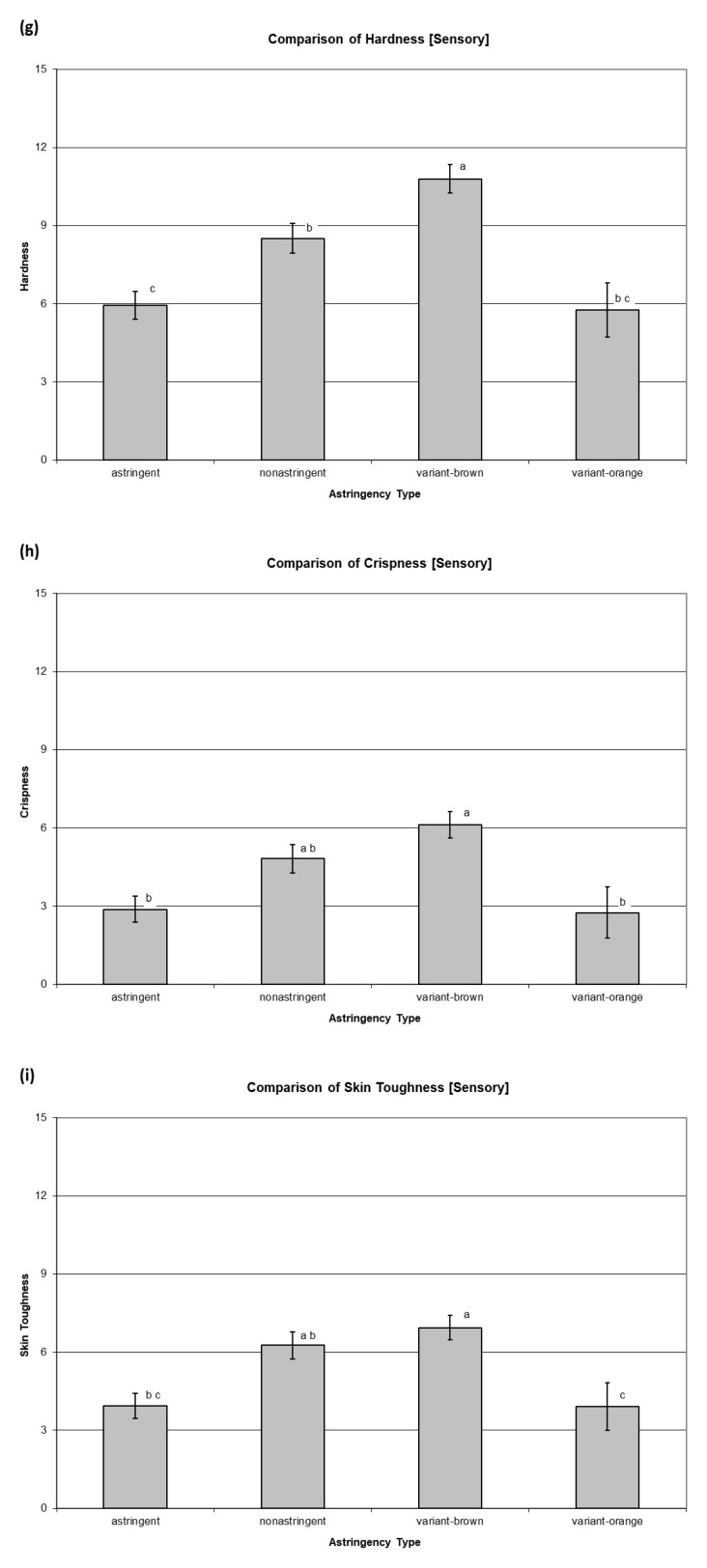

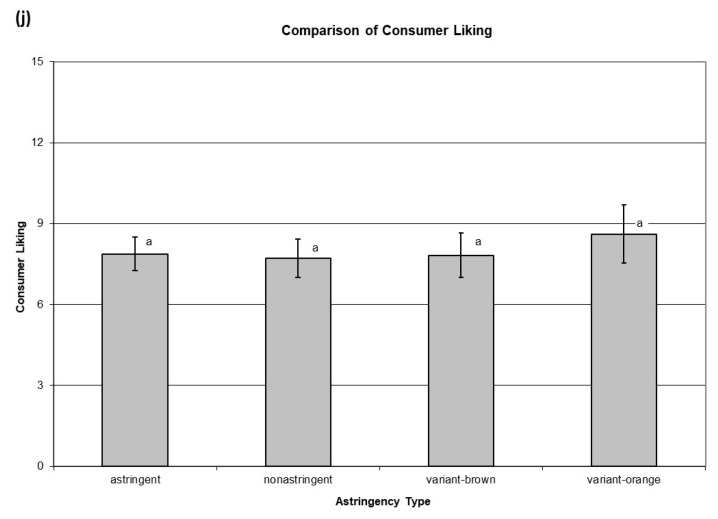
Comparison of Compressive Energy 1 (Texture Profile Analysis—TPA) (**a**), Cohesiveness (TPA) (**b**), Springiness (TPA) (**c**), Cutting Force (Shear) (**d**), Roughness (Sensory) (**e**), Moistness (Sensory) (**f**), Hardness (Sensory) (**g**), Crispness (Sensory) (**h**), Skin Toughness (Sensory) (**i**), and Consumer Liking (**j**) among the different persimmon astringency types, analyzed on an as-dried basis. Error bars represent +/− 1 standard error of the mean. Statistically significant differences at *p* < 0.001 are indicated with lowercase letters.

Texture analysis has also partially addressed the unique challenge of the diverse astringency types within the spectrum of available persimmon cultivars. Most instrumental measurements and sensory texture attributes were found to differ by astringency type, including the orange-versus-brown phenotype of the variant astringency type. See Table 7 for a summary of the ANOVA results and Supplemental Table S1 for a full listing of the ANOVA and Tukey’s Honestly Significant Difference (HSD) results. While the criterion for statistical significance was an ANOVA *p*-value less than 0.05, for brevity’s sake, Figure 1a–i show the mean values of only those attributes whose ANOVA results showed the strongest separation among the astringency types (*p* < 0.001). Dried astringent and variant-orange samples were statistically indistinguishable for all instrumental measurements and sensory texture attributes tested. The dried astringent and variant-orange samples generally exhibited relatively soft, smooth, and moist textures. Dried variant-brown samples exhibited hard and rough textures, and dried nonastringent samples fell in the middle. 

For other plant foods, the composition and morphology of the cell walls of the parenchyma cells are the microstructural features that drive the macro-texture of the food [14,27]. However, the dramatic textural variation among the dried forms of the different persimmon astringency types suggests that it is not the parenchyma cells but rather the tannin cells that most influence the texture of the dried product. Soluble tannins are the compounds that give persimmons their characteristic astringency; the insolubilization (aggregation) of the tannin molecules is the basis of natural deastringency processes in nonastringent and pollinated variant fruit as well as artificial deastringency treatments [47,48,49,50,51]. The tannins are located in specialized tannin cells that differ in size, morphology, and spatial distribution among the different astringency types [47,48,49,50]. In nonastringent cultivars, early during fruit development, the tannin cells stop growing [48,49] and the tannins insolubilize [49], such that the tannin cells are small and widely dispersed throughout the fruit tissue at harvest. For all other astringency types, the tannin cells continue to grow and remain densely connected as the fruit develops [47,48]. One can envision that, when the tannins inside these large and connected tannin cells begin to aggregate (insolubilize), a scaffold-like structure is formed. Indeed, the tannin cells around the seeds of variant fruit of the brown/nonastringent phenotype have been described as a “bundle in the flesh” [50]. While this structure apparently does not noticeably affect the texture of the fresh variant-brown fruit versus that of the fresh nonastringent fruit, the difference is greatly accentuated by the drying process, with dried variant-brown fruit having an essentially wood-like and highly unpalatable texture. Because the tannins of the astringent cultivars and variant-orange phenotypes of the variant cultivars remain soluble until harvest, no scaffold-like structure is formed, and the fruit remain relatively soft and pliable upon drying. Studies of the microstructure of the dried persimmon chips are recommended to confirm or refute this hypothesis for the mechanism behind the different textures of the astringent, nonastringent, variant-orange, and variant-brown persimmons.

While the persimmon chips exhibited striking differences in texture according to astringency type, mean consumer liking of the chips did not significantly differ by astringency type (Figure 1j). This is in agreement with our findings from the previous harvest season [1] and is a caution to persimmon growers not to assume that a certain persimmon cultivar will perform well or poorly in hot-air-dried form, based solely on the astringency type of the cultivar. Within the variant type, however, the orange (unpollinated) phenotype exhibited the apparently more-desirable texture traits (low Hardness, high Moistness, low Skin Toughness, low Fibrousness, etc.) than did the brown (pollinated) phenotype. This suggests that hot-air drying can be an appealing postharvest alternative for persimmon growers who unexpectedly find themselves with unpollinated variant fruit (i.e., unfit for sale as a fruit for immediate consumption) in a particular harvest.

An interesting case in the present study was the cultivar ‘Giombo’, whose first harvest was designated variant-orange but whose second harvest was variant-brown. (Seeds were present in the samples from both harvests, but presumably the brown phenotype had not had time to develop by the time of the first harvest.) Unfortunately, only the first harvest was evaluated by the consumer group, who gave it a relatively high mean liking score of 9.2. However, the instrumental and sensory measurements can fill in this knowledge gap. Applying the PLS model which incorporates both measurement types, the predicted liking score for the second harvest is 6.1. That is, the dried chips from the second harvest (brown phenotype) of this cultivar would have fared far worse than those from the first harvest (orange phenotype). Photographs of samples from the two harvests are shown in Supplemental Figure S1. 

Another interesting case is the cultivar ‘Nishimura Wase’, which had a very high consumer liking score of 10.2 despite being designated variant-brown. Of the 10 consumer panelists who gave ‘Nishimura Wase’ their top score, 6 described the interior flesh color as “orange”, 2 described it as having both orange and brown areas, 1 described it as “pale” (probably referring to orange), and 1 described it as “dark purple” (probably referring to brown). Thus, the highly preferred versions of the dried ‘Nishimura Wase’ samples predominantly exhibited the unpollinated/orange phenotype. 

#### 3.4.2. Comparison on Equal-a_W_ Basis

**Figure 2:**
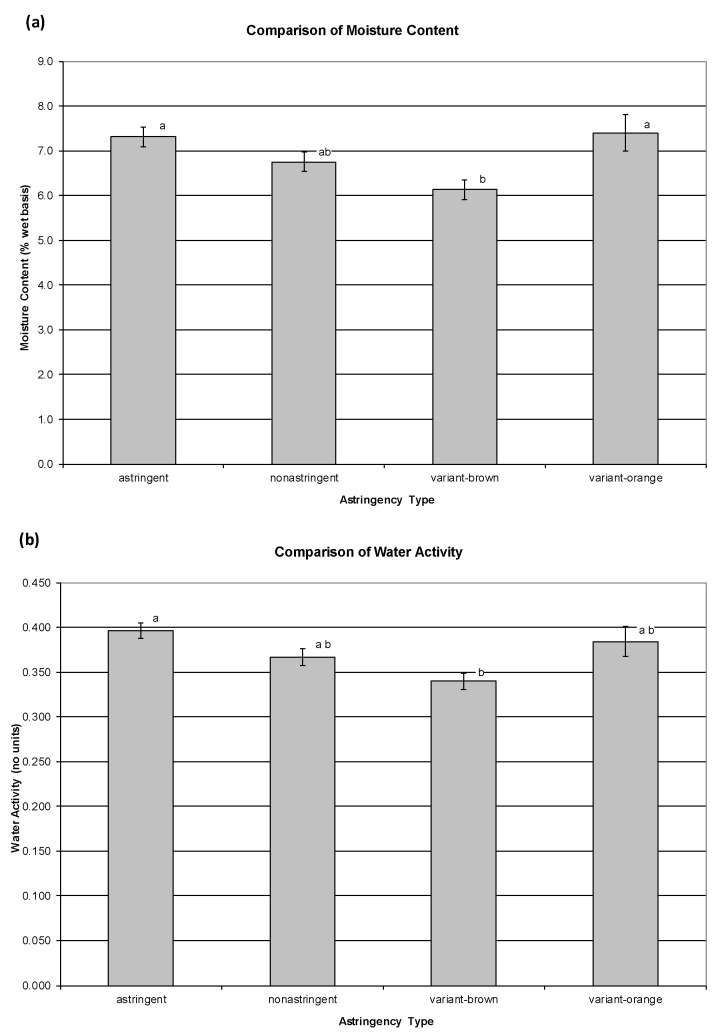
Comparison of moisture content (**a**) and water activity (a_W_) (**b**) among the different persimmon astringency types. Two high-moisture outlier samples (‘Nui Nai’ second harvest; ‘Mikatani Gosho’ third harvest) were excluded from this analysis. Error bars represent +/− 1 standard error of the mean. Statistically significant differences at *p* < 0.001 are indicated with lowercase letters.

In the preceding section, an as-dried basis was used for all analyses of the persimmon astringency types’ effects on the various texture attributes and consumer liking. That is, the persimmon samples in this study were physically evaluated at the moisture content/a_W_ that naturally resulted in the chips after the 52 °C/18 h drying process. As previously mentioned (Section 3.1), the moisture contents/a_W_s for all but 2 of the samples dried by the researchers fell within a fairly narrow band. Indeed, when an ANOVA was performed to determine the effect of astringency type on endpoint moisture content and a_W_, no significant differences were identified among the astringency types (moisture content *p*-value = 0.2020; a_W_ *p*-value = 0.1228). However, when the 2 unusually high-moisture samples (‘Nui Nai’ second harvest; ‘Mikatani Gosho’ third harvest) were excluded from the analysis, some systemic variation in moisture content/a_W_ among the astringency types was revealed. The *p*-value for the ANOVA for moisture content decreased to 0.0020, and the *p*-value for a_W_ decreased to 0.0005. Plots depicting the ANOVA and Tukey’s HSD results for moisture content and a_W_ (with the high-moisture outlier samples excluded) are given in Figure 2. It can be observed from Figure 2 that the hot-air-dried chips of astringent cultivars were significantly more moist and had higher a_W_ than the chips made from the variant-brown phenotype of variant cultivars. Nonastringent cultivars had intermediate values. The variant-orange phenotype cultivars had statistically similar values to the astringent cultivars, again showing some important similarities between these different astringency types. 

There is merit to comparing the persimmon astringency types on both an as-dried basis and on the basis of equal a_W_. Using an as-dried basis (as was done in the previous section), one can see the full effect of the persimmon astringency types, which apparently includes the different propensities of these types to hold onto moisture, even under harsh drying conditions. These differing hygroscopicities contribute to the overall texture of the product when the drying process is applied identically to all cultivars. A different set of insights can be gleaned from equilibrating the dried samples to the same moisture content or a_W_. This approach has been used in the past for analysis of the texture (and other quality indices) of dried apple chips [52]. Unfortunately, resource limitations dictated that this type of equilibration was not performed in the present study, so the associated physical data are not available. However, an in silico equilibration can be done by limiting the set of samples under consideration to only those within a narrow water activity range. This enables evaluation of the persimmon cultivars on a (nearly) equal-a_W_ basis and helps to isolate the effects of cultivar type on dried product texture, independent of a_W_. Thus, the analyses of Section 3.4.1 were re-run on 3 subsets of study samples: Low a_W_ (mean = 0.346, range = 0.333–0.357, *n* = 9), Medium a_W_ (mean = 0.375, range = 0.363–0.383, *n* = 8), and High a_W_ (mean = 0.400, range = 0.386–0.411, *n* = 9). These ranges were chosen to be as narrow as possible yet still include at least one variant-orange sample (of which there were only four in the overall sample set) and at least two samples of each of the other astringency types. In these analyses on an equal-a_W_ basis, far fewer texture attributes yielded significant ANOVA *p*-values than did the analyses on an as-dried basis. This is, in part, expected because of the lower number of samples in each set (8–9 vs. 47 in the original set) and the correspondingly lower discrimination power of the analysis. The ANOVA *p*-values are summarized in Table 8. In Table 8, if an attribute does not appear, then its ANOVA *p*-value was not significant for any of the 3 in silico a_W_s. The full table of ANOVA and Tukey’s HSD results is available as Supplementary Table S2. 

When the samples were considered on an equal-a_W_ basis, no attribute was significantly affected by astringency type consistently across all in silico a_W_ levels. This suggests that differences in texture attributes among the persimmon astringency types are sensitive to moisture level, with different attributes exhibiting differences at low, medium, and high a_W_, even within an already-narrow a_W_ range. The only attribute yielding significant ANOVA *p*-values for more than one in silico a_W_ was sensory Roughness. This indicates that Roughness may be the persimmon chip texture attribute that is least sensitive to moisture content. For the attributes Cohesiveness, Springiness, Roughness, and Hardness, the pattern of differences among the astringency types mirrored that found when the samples were earlier compared on an as-dried basis. That is, the variant-brown samples were found to have negative texture attributes: low Cohesiveness, low Springiness, high Roughness, and high Hardness. Also as before, the astringent and variant-orange samples were statistically indistinguishable for these attributes. Thus, the poor performance of the variant-brown samples in dried form and the similar textural features of the variant-orange and astringent cultivars seem to be phenomena that are moisture-independent at certain moisture levels. At the 0.400 (High) a_W_ level, the significant difference among astringency types for the attribute of Tooth Packing is intriguing. The variant-orange and nonastringent samples had the highest Tooth Packing, the astringent samples had the lowest, and the variant-brown were in the middle. This pattern of statistical similarity was not observed for other analyses in this work, and Tooth Packing did not differ by astringency type at the Low and Medium in silico a_W_ levels. So, this phenomenon merits further investigation but may simply be a result of the low n in the equal-a_W_ subsets. The final point of interest in this equal-a_W_ analysis is that consumer liking of the persimmon chip product is not significantly affected by astringency type (bottom row of Table 8). So, the recommendation to avoid ruling-in or -out any particular cultivar for drying solely based on its astringency type holds whether the analysis is done on an as-dried basis or on an equal-a_W_ basis. 

## 4. Conclusions

Models of fair-to-good quality can be used to predict all of the sensory texture attributes of dried persimmon chips—except Tooth Packing—from the instrumental techniques of TPA and shear test. This finding will reduce the time necessary to characterize the texture of persimmon chips and perhaps similar dried-fruit products. PLS modeling has identified the texture attributes most desired by consumers for this product, and the preference is for a moist, smooth texture rather than the dry, crispy texture that was originally intended. Identification of persimmon cultivars that possess the desirable texture traits can be done based on this work, and astringency type should not be used a priori to screen in or out any cultivars. Additionally, the multivariate modeling approach used in this work has enabled a comparison of instrumental- and sensory-panel-assessed texture attributes; some potential mismatches have been identified between the metrics that the instruments are intended to measure and those that the trained sensory panel reports. These present opportunities for refinement of the instrumental measurements to better match texture perception by humans. Lastly, this work has generated some hypotheses about how tannin-cell-driven microstructural differences among astringent, nonastringent, and variant persimmon cultivars may contribute to dramatic macro-texture differences in the hot-air-dried forms of these fruit. 

## Figures and Tables

**Table 1:** Consumer evaluation statuses, number of harvests, astringency types, and sources of the persimmon cultivars in the present study.

Cultivar	Evaluated by Consumer Panel ^1^	Number of Harvests	Astringency Type	Source
Akoumanzaki	+	1	V-B	R
Chienting		1	V-B	R
Chocolate		1	V-B	C-N
Costata	+	1	A	R
Farmacista Honorati	+	1	V-O	R
Fuji	+	1	A	R
Fujiwaragosho		1	V-B	R
Fuyu Imoto	+	1	N	C-N
Fuyu Jiro		1	N	C-N
Giant Fuyu		1	N	C-N
Giombo	+	2	V-O (1st), V-N (2nd)	R
Gofu	+	2	V-O (both harvests)	R
Guang Yang	+	1	N	R
Hachiya	+	1	A	C-N
Hazegosho	+	2	N	R
Ichidagaki		1	A	R
Izu		1	N	C-N
Kakiyamagaki	+	1	A	R
Korean		1	A	R
Lampadina		1	V-B	R
Lycopersicon		1	A	R
Mandarino		1	V-B	R
Maru		1	V-B	C-N
Matsumoto Wase Fuyu	+	2	N	R
Matsumoto Wase Fuyu	+	1	N	C-N
Mikatani Gosho	+	3	V-B (all 3 harvests)	R
Nishimura Wase	+	1	V-B	C-N
Nui Nai	+	2	A	R
Okugosho	+	1	N	R
Rispoli	+	1	V-B	R
Rose Yanka	+	2	A	R
Saijo	+	1	A	C-N
Sangokuichi		1	V-B	R
Suruga	+	2	N	R
Tanenashi	+	1	A	C-N
Thiene	+	1	V-B	R
Tishihtzu	+	2	A	R
(grocery store)	+	unknown	unknown	C-S

^1^ “+” in the consumer panel column indicates that the sample was included in the consumer hedonic rank-rating portion of the study. (All samples were evaluated by the trained sensory panel and by instrumental measurements. See the *Consumer Test* section for additional explanation.) For cultivars with multiple harvests, only the first harvest of the cultivar was used for consumer evaluation. Astringency Type: A = astringent, N = nonastringent, V-B = variant with brown/pollinated phenotype, V-O = variant with orange/unpollinated phenotype. Source: R = research plot, C-N = commercial nursery, C-S = commercial retail store.

**Table 2:** Instrumental measurements used to characterize persimmon chips.

Attribute	Method	Definition	Units
Hardness 1	TPA	Maximum force applied to the sample during the first compression	N
Compressive Energy 1	TPA	Area under the curve for the first compression	N*mm
Hardness 2	TPA	Maximum force applied to the sample during the second compression	N
Compressive Energy 2	TPA	Area under the curve for the second compression	N*mm
Cohesiveness	TPA	Ratio of the area under the curve for the second compression to that under the curve for the first compression	(unitless)
Springiness	TPA	Ratio of the duration of contact with the sample during the second compression to that during the first compression	(unitless)
Chewiness	TPA	Mathematical product of Hardness 1, Cohesiveness, and Springiness	N
Resilience	TPA	Upstroke energy of the first compression divided by the downstroke energy of the first compression	(unitless)
Cutting Force	Shear	Maximum force applied to the sample during the shearing	N
Cutting Energy	Shear	Area under the curve for the shearing	N*mm
Peak Count	Shear	Number of minor peaks before the Cutting Force is reached	(unitless)

TPA = Texture Profile Analysis.

**Table 3:** Sensory attributes used to characterize persimmon chips.

Attribute	Definition	Location on 15 cm Unstructured Line Scale, Standard, Description of Standard	Brand/Manufacturer (When Applicable)
Chewiness	number of chews required for a standard-sized piece before the product is swallowed	0.0 white bread fresh, center cut, inch cube	Wonder/Flowers Foods (Thomasville, Georgia)
		7.5 licorice candy 1 piece	Red Vines/American Licorice Company (La Porte, Indiana)
		15.0 chewy chocolate candy midget size, 1 piece	Tootsie Rolls/Tootsie Roll Industries (Chicago, Illinois)
Crispness	noise and force with which the sample breaks or fractures when bitten through with the incisors	2.0 granola bar ¼ bar	Quaker Chewy Chocolate Chip/PepsiCo (Chicago, Illinois)
		7.0 oat cereal 1 oz.	Cheerios/General Mills (Minneapolis, Minnesota)
		15.0 Melba toast 1 cracker	Old London/B&G Foods, Inc. (Parsippany, New Jersey)
Fibrousness	amount of long, stringy particles between teeth during chew	0.0 banana ripe, fresh, inch slice	-
		7.5 pineapple ripe, fresh, tissue next to skin, inch cube	-
		15.0 artichoke leaf from canned artichoke hearts, 3 leaves	Trader Joe’s (Monrovia, California)
Hardness	force required to compress the sample between the incisors	1.0 cream cheese regular, inch cube	Philadelphia/The Kraft Heinz Company (Chicago, Illinois)
		7.0 hot dog microwave-heated for 30 sec., inch slice	Oscar Mayer Classic Wieners/Kraft Heinz Company (Chicago, Illinois)
		14.5 hard candy 2 pieces, one color	LifeSavers/Mars, Inc. (Chicago, Illinois)
Moistness	amount of wetness or oiliness (moistness if both) on surface	0.0 unsalted cracker 1 cracker	Nabisco Premium/Mondelēz Intl. (Deerfield, Illinois)
		15.0 apple Red Delicious, uncooked, freshly sliced, inch slice	-
Roughness	overall amount of small and large particles on the surface	0.0 gelatin dessert 2 tsp.	Snack Pack Juicy Gels/ConAgra Foods (Omaha, Nebraska)
		8.0 potato chips 2 pieces	Pringles/Kellogg Company (Battle Creek, Michigan)
		15.0 rye wafer ½ wafer	Wasa/Wasa North America LLC (Northbrook, Illinois)
Tooth Packing	amount of product left on teeth after expectoration	0.0 mini clams 3 pieces	Chicken of the Sea/Thai Union Group (Samutsakorn, Thailand)
		7.5 Graham cracker inch square	Nabisco Honey Maid/Mondelēz Intl. (Deerfield, Illinois)
		15.0 chewy fruit candy 1 piece	Jujyfruits/Ferrara Candy Company (Oakbrook Terrace, Illinois)
Toughness of Skin	force required to bite through the skin with the incisors	1.0 dates fancy grade, 2 pieces	Medjool/Trader Joe’s (Monrovia, California)
		15.0 summer sausage casing intact, ¼ inch slice	Hillshire Farm/Tyson Foods, Inc. (Springdale, Arkansas)

**Table 4:** Predictions of sensory attributes from instrumental measurements using partial least-squares (PLS) models. Sensory attributes are listed in the order in which they were evaluated by the sensory panelists. Underlined font highlights Variable Importance on the Projection (VIP) scores greater than 1.2.

	Model Diagnostics			VIP Scores by Instrumental Measurement										
				TPA								Shear		
Sensory Attribute	Cross- Validation RMSE	Adjusted R^2^ of Predicted vs. Actual	Number of Latent Factors	Hardness 1	Compressive Energy 1	Hardness 2	Compressive Energy 2	Cohesive- ness	Springi- ness	Chewi- ness	Resilience	Cutting Force	Cutting Energy	Peak Count
Roughness	0.727	0.595	4	1.038	1.324	0.965	1.183	0.773	0.959	0.696	1.303	1.309	1.106	1.357
Moistness	0.800	0.481	3	1.089	1.311	0.962	1.240	0.767	1.109	0.583	0.671	1.567	1.433	1.389
Hardness	0.605	0.715	3	1.152	1.304	1.136	1.304	0.506	0.472	0.574	0.698	1.187	0.953	1.537
Crispness	0.589	0.742	3	1.096	1.186	1.093	1.239	0.387	0.396	0.702	0.259	1.105	0.777	2.052
Skin Toughness	0.728	0.613	3	1.192	1.303	1.131	1.281	0.460	0.423	0.617	0.437	1.304	1.171	1.684
Fibrousness	0.808	0.483	2	1.166	1.135	1.184	1.361	0.631	0.674	0.992	0.278	1.070	1.384	1.142
Chewiness	0.673	0.656	3	1.196	1.292	1.228	1.306	0.383	0.428	0.972	1.375	1.326	1.081	0.935
Tooth Packing	0.977	0.125	1	1.128	0.816	1.283	1.180	0.369	0.534	1.044	0.074	1.424	1.103	1.624

RMSE = Root-Mean-Square Error.

**Table 5:** Predictions of consumer liking score from sensory attributes and instrumental measurements using partial least-squares (PLS) models. Underlined font highlights Variable Importance on the Projection (VIP) scores greater than 1.2.

	**Model Diagnostics**			**VIP Scores by Measurement or Attribute**										
				**Instrumental—TPA**								**Instrumental—Shear**		
**Model**	**Cross-** **Validation RMSE**	**Adjusted R^2^ of Predicted vs. Actual**	**Number of Latent Factors**	**Hardness 1**	**Compressive Energy 1**	**Hardness 2**	**Compressive Energy 2**	**Cohesiveness**	**Springiness**	**Chewiness**	**Resilience**	**Cutting Force**	**Cutting Energy**	**Peak Count**
Instrumental & Sensory	0.878	0.372	1	0.127	0.199	0.600	0.681	0.624	0.176	0.323	0.109	0.659	0.232	1.424
Instrumental Only	1.062	0.415	3	1.014	0.698	0.874	1.039	1.023	1.104	0.699	1.662	1.329	1.404	2.647
Sensory Only	0.798	0.523	2	-	-	-	-	-	-	-	-	-	-	-
				**VIP Scores by Measurement or Attribute (continued)**										
				**Sensory**										
		**Model**		Rough- ness	Moistness	Hardness	Crispness	Skin Toughness	Fibrousness	Chewiness	Tooth Packing			
		Instrumental & Sensory		1.094	0.720	1.613	1.299	1.317	1.731	1.786	1.591			
		Instrumental Only		-	-	-	-	-	-	-	-			
		Sensory Only		0.689	1.082	0.985	0.859	0.888	1.204	1.466	1.060			

RMSE = Root-Mean-Square Error; VIP = Variable Importance on the Projection; TPA = Texture Profile Analysis.

**Table 6:** Predictions of consumer liking score from sensory attributes and instrumental measurements using ordinary least-squares models. “Sparse” models include those attributes whose Variable Importance in Projection (VIP) scores were greater than 1.2 for the original complete partial least-squares models.

			**Model Coefficients**					
	**Model Diagnostics**			**Instrumental—TPA**	**Instrumental—Shear**			
**Model**	**RMSE of Predicted vs. Actual**	**Adjusted R^2^ of Predicted vs. Actual**	**Constant**	**Resilience**	**Cutting Force**	**Cutting Energy**	**Peak Count**	
Instrumental & Sensory—Sparse	0.598	0.432	15.71	-	-	-	−0.12	
Instrumental Only—Sparse	1.596	0.219	9.10	2.94	−0.04	0.01	−0.17	
Sensory Only—Sparse	1.367	0.427	12.68	-	-	-	-	
Shear Peak Count Only	1.606	0.208	9.94	-	-	-	−0.16	
			**Model Coefficients (continued)**					
			**Sensory**					
	**Model**		**Hardness**	**Crispness**	**Skin Toughness**	**Fibrousness**	**Chewiness**	**Tooth Packing**
	Instrumental & Sensory—Sparse		−0.53	0.66	0.31	−0.51	−0.24	0.40
	Instrumental Only—Sparse		-	-	-	-	-	-
	Sensory Only—Sparse		-	-	-	−0.58	−0.57	-
	Shear Peak Count Only		-	-	-	-	-	-

RMSE = Root-Mean-Square Error; TPA = Texture Profile Analysis.

**Table 7:** Summary of Analysis of Variance (ANOVA) results for effect of astringency type on instrumental texture attributes, sensory texture attributes, and consumer liking.

	**Instrumental—TPA**							
	**Hardness 1**	**Compressive Energy 1**	**Hardness 2**	**Compressive Energy 2**	**Cohesiveness**	**Springiness**	**Chewiness**	**Resilience**
F ratio	3.191	9.423	2.708	4.011	6.709	10.989	0.260	4.358
Probability > F	0.033	<0.001	0.057	0.013	<0.001	<0.001	0.854	0.009
	**Instrumental—Shear**							
	**Cutting Force**	**Cutting Energy**	**Peak Count**					
F ratio	7.411	5.599	1.334					
Probability > F	<0.001	0.003	0.276					
	**Sensory (Trained Panel)**							
	**Roughness**	**Moistness**	**Hardness**	**Crispness**	**Skin Toughness**	**Fibrousness**	**Chewiness**	**Tooth Packing**
F ratio	31.794	16.887	15.559	8.153	8.395	4.959	2.398	3.176
Probability > F	<0.001	<0.001	<0.001	<0.001	<0.001	0.005	0.081	0.034
	**Consumer Liking**							
F ratio	0.173							
Probability > F	0.913							

**Table 8:** Summary of Analysis of Variance (ANOVA) results (probability > *F* values/*p*-values) for effect of astringency type on selected instrumental texture attributes, sensory texture attributes, and consumer liking, analyzed on an equal-a_W_ basis. *p*-values less than 0.05 are highlighted in bold font.

	In Silico a_W_		
Attribute	0.346 (Low)	0.375 (Medium)	0.400 (High)
Cohesiveness (TPA)	**0.0134**	0.1498	0.3271
Springiness (TPA)	**0.0038**	0.3589	0.2559
Roughness (sensory)	**0.0078**	0.1364	**0.0227**
Hardness (sensory)	0.1785	**0.0460**	0.5848
Tooth Packing (sensory)	0.2465	0.5899	**0.0078**
Consumer Liking	0.8900	0.3529	0.6744

**Figure A1:**
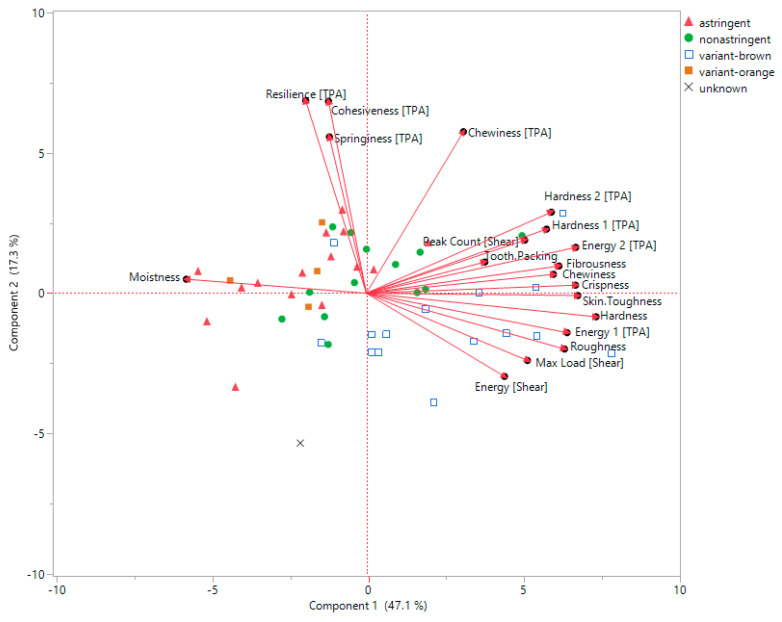
Principal components analysis biplot for the instrumental and trained sensory panel texture variables. Instrumental texture variable labels are followed by bracketed text indicating from which method they are derived (TPA = Texture Profile Analysis). Sensory texture variable labels have no bracketed text.

## Supplementary Materials

The following are available online at https://www.mdpi.com/2304-8158/9/10/1434/s1, File S1: Consumer persimmon chip rank-rating scoresheet; Table S1: Analysis of Variance (ANOVA) and Tukey’s Honestly Significant Different (HSD) results for effect of astringency type on instrumental texture attributes, sensory texture attributes, and consumer liking; Table S2: Analysis of Variance (ANOVA) and Tukey’s Honestly Significant Different (HSD) results for effect of astringency type on instrumental texture attributes, sensory texture attributes, and consumer liking when samples are compared on an equal-water activity basis; Figure S1: First (A) and second (B) harvests of cultivar “Giombo”, exhibiting the unpollinated/orange and pollinated/brown phenotypes, respectively.

## Appendix A

### Principal Components Analysis (PCA) of Instrumental and Trained Sensory Panel Texture Variables

The primary aim of this study was to provide persimmon growers and processors with actionable information about how the texture of dried persimmon products affects consumer preference. However, some readers may be interested in additional detail about the relationships among the instrumental and sensory texture variables. Thus, a principal components analysis (PCA) was performed on the data set used for this study.

The first two principal components described a cumulative 64.4% of the variation in the instrumental and trained sensory panel data. The scree plot did not exhibit an obvious inflection point, so the first two principal components were selected for further examination. A biplot of the first two components is shown in Figure A1. Loadings are depicted as red vectors, and scores are color- and shape-coded by the cultivar astringency type (or variant phenotype). The unknown-type score is for the store-bought (already dried) sample.

There are a number of features of interest in the PCA biplot:

- The first principal component (PC1) is driven primarily by the dichotomy of sensory Moistness versus the majority of the other variables. Thus, for this product, Moistness is mutually exclusive with the other variables, with the exceptions of TPA Resilience, Cohesiveness, and Springiness, which have small absolute-value loadings on PC1. The latter TPA attributes, along with TPA Chewiness are strongly positively loaded on the second principal component (PC2).
- The sensory attribute of Tooth Packing has the smallest loading (shortest vector) of all the variables. This is in agreement with our finding from PLS that Tooth Packing could not be well-predicted by the instrumental variables. This is because Tooth Packing is evaluated post-expectoration, and the physical setup of the instrumental texture measurements does not simulate this situation.
- As was also observed in the PLS regression analysis, TPA Hardness (both 1 and 2) is not closely correlated with sensory Hardness. Rather, the instrumental measurement most closely correlated with sensory Hardness is TPA Energy 1.
- Although they are in the same quadrant of the biplot, TPA Chewiness and sensory Chewiness are not particularly close to each other; there are multiple other instrumental measurements that are closer to sensory Chewiness than is TPA Chewiness. This suggests that (for this product, at least) there is a mismatch between what the TPA protocol is measuring for Chewiness versus what the sensory panelists are evaluating for that same term.
- The scores of the variant cultivars exhibiting the orange phenotype (solid orange squares in Figure A1) clustered closely with the scores of the astringent cultivars (solid red triangles), suggesting that the microstructures of these fruit are very similar, despite their being from nominally-different astringency types.
- The biplot shows quite clearly how the hot-air-dried chips made from the variant cultivars exhibiting the brown phenotype (open blue squares) were prone to having hard, tough, rough, and otherwise generally undesirable texture traits. This emphasizes the point that the variant-brown fruit—while highly palatable in fresh form—should not be used for hot-air-drying.
- The score for the store-bought sample (black ‘X’) is quite off by itself in the lower right-hand quadrant of the biplot. This indicates that this sample was dried under very different conditions than the other samples, was a cultivar not explored in the rest of this study, experienced different storage conditions from fall 2016 to spring 2017, or some combination of these circumstances.

Additional observations can be made by the interested reader; the underlying dataset is available from the authors upon request.
