# Supplementary material for: Texture of Hot-Air-Dried Persimmon (Diospyros kaki) Chips: Instrumental, Sensory, and Consumer Input for Product Development
Source: Foods. 2020 Oct 10;9(10):1434. doi: 10.3390/foods9101434 (PMC7601633; doi:10.3390/foods9101434)
Supplement: Supplementary file 1 [file foods-09-01434-s001.zip › File S1.docx]

**DRIED PERSIMMON SENSORY EVALUATION**

1. Check to make sure that you received the following materials in your bag:
   - Five black sample cups with samples
   - A strip of stickers with 5 sample codes that match your sample cups
   - Evaluation form (this sheet)
2. Please read through these instructions completely **BEFORE** beginning evaluation of the samples.
3. Please taste the samples, one at a time, in the order designated by the strip of stickers (left to right). You may re-taste once you have tasted **all FIVE samples once**.
4. Rate the samples according to how much you like them, by placing the sticker with the corresponding sample number in the boxes along the scale below.
5. Turn the sheet over and complete the questions on the back side of this evaluation form.

***DISLIKE***

***LIKE***


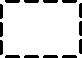

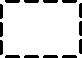

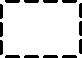

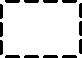

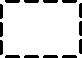

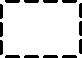

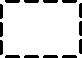

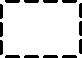

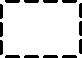

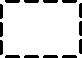

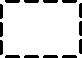

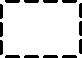

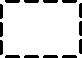

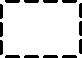

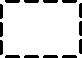


**NEITHER DISLIKE NOR LIKE**

**Please make sure to complete the questions on the other side of this sheet… ~~~~~~~~~>**

Now, please tell us a little more about the sample you liked the **MOST**.

First, copy that sample’s number here: __________

What color was the flesh (inside) of that sample? ____________________

What color was the skin (outside) of that sample? ____________________

How would you describe the texture of that sample? __________________________________

How would you describe the flavor of that sample? ___________________________________

If a package of that sample cost the same as a package of banana chips, how likely would you be to buy a package of that sample? (mark your answer by placing a vertical line on the scale below)

Definitely **Would Not** Buy

Definitely **Would** Buy

How often do you consume FRESH persimmons during the season (~September – December)?

☐ daily

☐ 3-5 times/week

☐ 1-2 times/week

☐ 1-3 times/month

☐ less than once a month

☐ never

How often do consume dried fruit, ON ITS OWN (ie. not in trail mix, cereal, etc.)?

☐ daily

☐ 3-5 times/week

☐ 1-2 times/week

☐ 1-3 times/month

☐ less than once a month

☐ never

Which of the following aspects of the dried persimmon samples influenced you to make the choices you did? Please check all that apply.

☐ Appearance

☐ Texture

☐ Flavor

☐ Other, please specify_________________

Did any of the samples stand out, either positively or negatively? Please explain:

__________________________________________________________________________________________________________________________________________________________________________________________________________________________________________

What is your age?

☐ 10-18 years old

☐ 18-29 years old

☐ 30-39 years old

☐ 40-49 years old

☐ 50-59 years old

☐ 60-69 years old

☐ 70 years and over

Which of the following best describes you?

☐ Male

☐ Female

**Thank you very much for your participation!**
