# Supplementary figures and images for: Texture of Hot-Air-Dried Persimmon (Diospyros kaki) Chips: Instrumental, Sensory, and Consumer Input for Product Development
Source: Foods. 2020 Oct 10;9(10):1434. doi: 10.3390/foods9101434 (PMC7601633; doi:10.3390/foods9101434)

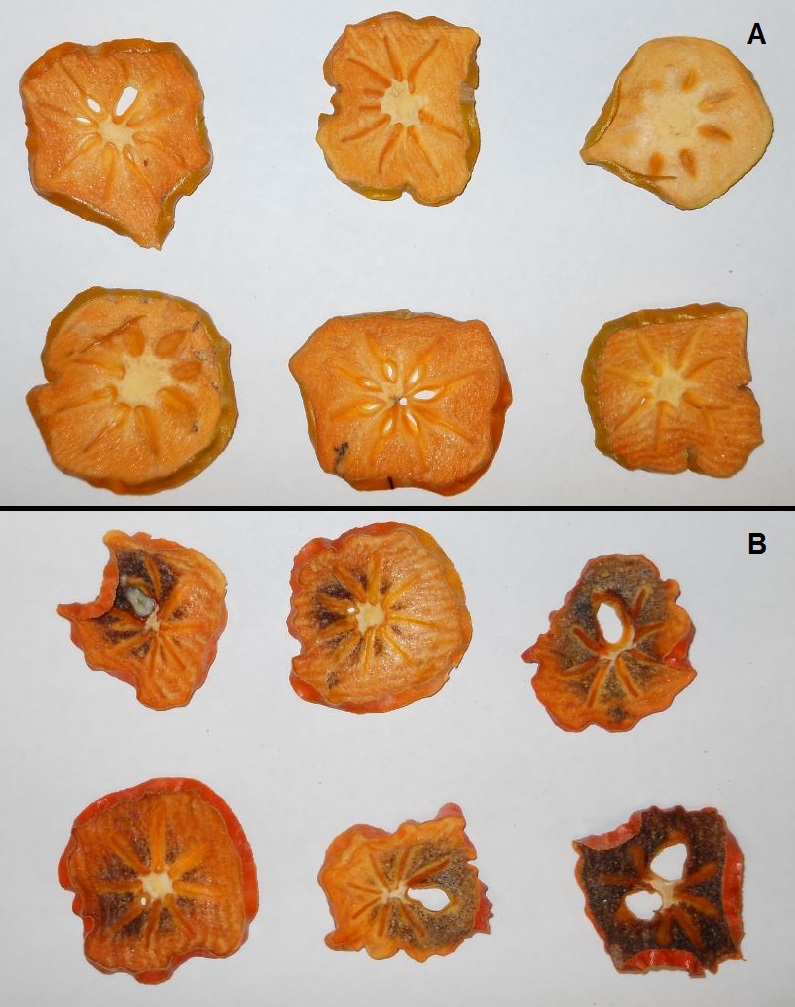

Supplement: Supplementary file 1 [file foods-09-01434-s001.zip › Figure S1.JPG]
